# Hospital Finance and Economy

**Published:** 1888-10-13

**Authors:** 


### HOSPITAL FINANCE AND ECONOMY.—II.

BY A MEDICAL SUPERINTENDENT.

*(Continued from p. 336, Vol. IV.)*

PURSUING our inquiries into Hospital Finance, from the statistics published at the instance of the Hospital Sunday Fund, we find that the Council distributed its awards to various institutions, small and large, situated within and without the metropolitan area, but which can scarcely be classified either with the general or special hospitals. These mainly consist of Cottage Hospitals, Invalid Asylums of various descriptions, Convalescent Homes, and Dispensaries. Some of the establishments are placed at considerable distances from the metropolis, but as they unquestionably derive their chief supply of inmates from London, there can be no doubt of the expediency of their participating in the bounty of the fund. In analysing the cost of the beds in these institutions we are fortunately not embarrassed with the question of out-patients, but while making every allowance for the peculiar conditions under which each is maintained, such as the smallness of the establishments and the necessity for retaining in each a permanent staff, it is impossible to reconcile the great differences which exist in their expenditure when compared with the work accomplished. By their own figures the authorities of these institutions confess to an expenditure which varies from a minimum of eight shillings and a halfpenny for the maintenance of a bed per week, to a maximum of £4 15s. 1*d.*, but we will take the establishments in order, and attempt to analyse the annual cost per head in each.

| | Average No. of Patients. | Cost per Head per Annum. | Amount Paid Over by Hospital Fund. |
|---|---|---|---|
| Beckenham Cottage Hospital | 6 | £83 | £47 |
| Blackheath and Charlton Hospital | 7 | 120 | 68 |
| Chislehurst and Cray Valley Hospital | 6 | 90 | 30 |
| Eltham Hospital | 3 | 111 | 36 |
| Enfield | 4 | 69 | 36 |
| Epsom and Ewell | 6 | 82 | 42 |
| Reigate and Redhill Cottage Hospital | 13 | 68 | 52 |
| Shedfield Cottage Hospital | 5 | 46 | 10 |
| Sidcup Cottage Hospital | 4 | 76 | 2 |
| Wimbledon Cottage Hospital | 7 | 64 | 36 |
| Establishment for Gentlewomen, Harley Street | 10 | 239 | 83 |
| Hampstead Home Hospital | 9 | 133 | 52 |
| National Sanatorium, Bournemouth | 35 | 60 | 73 |
| Sea-Bathing Infirmary, Margate | 162 | 52 | 573 |
| Invalid Asylum, Stoke Newington | 20 | 50 | 73 |
| Firs Home, Bournemouth | 16 | 79 | 21 |
| St. Catherine's Home, Ventnor | 10 | 119 | 47 |

One cannot conceive why the highest award but one in the above list should have been given to the costliest establishment, unless on the supposition that the expenditure for the year included the whole cost of the building, which is extremely unlikely. Moreover, the patients' payments average nearly £1,000, a sum equivalent to £100 a-year for each occupied bed. It would greatly simplify matters if a column in the statistical tables were set apart for extraordinary expenditure which might occur in the course of the year in the shape of new buildings or extensions. Such additions are almost invariably made from special funds, and ought not to be included in the ordinary expenditure of the institution, otherwise our estimated value of the occupied bed is worthless. Of course, ordinary repairs, the introduction of new and improved machinery, and petty structural alterations are being constantly carried out in most hospitals, and are everywhere recognized as legitimate and ordinary expenditure; but these ought not to be mixed up with new and expensive additions. Mr. Quennell has justly drawn attention to this fact in his remarks on our estimate of the annual cost at the Westminster Hospital, which has long been known to experts as one of the most economically-conducted institution of its kind in London; but there are few hospitals which can boast of having built a chapel and a new medical school and added them on to the parent establishment in the course of the same year.

We will now take the case of the Convalescent Homes which participate in the benefits of the Sunday Fund. These, 18 in number, give a very inadequate idea of the extent to which this species of curative agency is carried out in the suburbs of London or at the seaside, as several of the larger institutions do not sue for Hospital Sunday relief at all, while a large contingent of small Homes are maintained at the expense of private individuals, supplemented in most cases by payments from the patients, ranging from 2*s.* 6*d.* to £1 1*s.* a-week. The principle of self-help as illustrated by the support given to these valuable institution, which may be fairly considered an adjunct to the hospitals, is very gratifying, and discloses a source of revenue which, but for the supineness of the authorities, might be introduced with advantage into every voluntarily-supported hospital in the country. It is equally gratifying to find that the convalescent homes as a rule are managed in an economical and efficient manner, notwithstanding the fact that many of them are either closed altogether in winter or have their inmates greatly reduced in number. The causes which have given rise to a high average cost in several may possibly be thus explained, or, as in the case of the Scarlet Fever Home, by the sanitary requirements of this excellent institution. It consists with our knowledge also, that the Metropolitan Convalescent Asylum, the oldest and by far the largest in the country, was closed for necessary repairs during a considerable part of last year, which circumstance, while increasing the cost of the occupied beds above the average of former years, has diminished the cost of the branch establishment at Bexhill-on-Sea, from the increased pressure put on its accommodation. Why a bed in a small institution at Hampton Court should cost nine times as much as one in a home at Epping Forest is not evident, though it may satisfy those associated with its management, and it has been considered eligible for an award; but while we do not presume to call in question the *needs* of such establishments, it must be manifest to outsiders that their *merits* are conspicuously small.

The convalescent hospitals are here given in the order in which they appear in the report of the Committee of Distribution of the Council of the Hospital Sunday Fund for 1888:—

| | Average No. of Beds occupied. | Cost of each per annum. | Award. |
|---|---|---|---|
| Metropolitan Convalescent Institution | 147 | £47 | £656 |
| Bexhill Seaside Convalescent Home | 75 | 25 | 161 |
| All Saints' Convalescent Home, Eastbourne | 248 | 25 | 599 |
| Mrs. Gladstone's Convalescent Home, Woodford | 76 | 21 | 208 |
| Hanwell Convalescent Home, Middlesex | 18 | 37 | 26 |
| Herbert Convalescent Home, Bournemouth | 26 | 44 | 21 |
| King's College Convalescent Home, Hemel Hemptstead | 32 | 61 | 125 |
| Mrs. Kitto's Convalescent Home, Reigate | 18 | 31 | 73 |
| Mrs. Marshman's Convalescent Home, Brighton | 108 | 33 | 83 |
| Mary Wardell Convalescent Home for Scarlet Fever | 23 | 81 | 121 |
| Morley Seaside Convalescent Home, St. Margaret's | 20 | 36 | 52 |
| St. Andrew's Convalescent Home, Clewer | 75 | 45 | 146 |
| St. Andrew's Convalescent Home, Folkestone | 90 | 33 | 146 |

| | Average No of Beds occupied. | Cost of each per annum. | Award. |
|---|---|---|---|
| St. Leonards-on-Sea Convalescent Home for Children | 66 | 22 | 104 |
| St. Mary Magdalene Convalescent Home, Paddington | 30 | 28 | 52 |
| Seaford Convalescent Home | 50 | 61 | 104 |
| Bexhill and Ramsgate Convalescent Home for Children | 11 | 65 | 53 |
| Princess Frederica's Convalescent Home, East Molesey | 5 | 189 | 42 |

Of the four well-known lying-in hospitals, only three have applied for help from the Sunday Fund, the City of London from some cause having been omitted from the list.

In attempting to analyse the expenditure of these institutions, it would be manifestly unfair to judge them by the principle adopted with other curative establishments, since much of their work is carried on at the residences of the patients by midwives and others, whose service are entitled to remuneration.

In the three establishments participating, the cost of in-patients, as stated by the several authorities, varies from £91 per head per annum at Queen Charlotte's, to £150 at the General Lying-In, while an out-patient (we presume a confinement) at the latter institution costs no more than 5*s.*—at the British Lying-in it is increased to double that amount. The gross annual expenditure of the three charities is £8,428, and the joint awards this year amounted to £469, of which the hospital in the Marylebone Road received the lion's share, which, no doubt, it deserved. It is important to notice the character of the work done by these separate establishments. The British Lying-in attends to four times the number of confinements outside the hospital that it does inside, the General Lying-in to twice the number, while Queen Charlotte's maintains almost as many in the hospital as it attends to at their own homes.

We lastly arrive at the dispensaries, 50 in number, which participate to a small extent in the benefits of the Hospital Sunday Fund. Sixteen of these are entered as provident, by which it is assumed that they are managed on a co-operative principle, the shareholders paying weekly, monthly, or annual premiums during health, in order that they may have medical aid in sickness. But as there are only fourteen entered on the list in which payments from the patients are not received, while sums varying from £16 to £636 have been contributed by the patients to others not specially entered as provident, it may be assumed that 36 out of the 50 dispensaries partake more or less of a provident character. It would follow, then, that on account of the principle on which the awards are based more than two-thirds of the number have to suffer in some degree from their self-supporting or partially self-supporting policy, a circumstance much to be regretted both on account of the recipients of the charities as well as of the medical men who administer them. With regard to this administration, combined with establishment charges, it is not easy, as was the case with the hospitals, to reconcile the differences which appear to exist between the money values of the treatment pursued at the different dispensaries, and without a more defined knowledge of the circumstances of each, it would be hazardous to express an opinion on their respective needs and merits. In a column of the statistics, so often referred to, the dispensary authorities give the average cost of patients at their respective institutions, from which we gather the following facts: Patients cannot be treated for less than 1*s.* a-head, and then only at an East-end dispensary. The maximum cost is 8*s.* 6½*d.* a-head at a dispensary in Upper Norwood, non-provident in the strict application of the term; while the expenses of a midwifery case are calculated at 5*s.* at the Western, the Belgrave, and the Farringdon dispensaries, and £1 1*s.* at the Notting Hill, Hampstead, and St. John's Wood dispensaries, the three last-named being classified as provident. At the Royal Maternity Charity—the largest of its kind in this and probably in any other country—the cost of each confinement is reckoned at 10*s.* a-head, and as these average 4,000 annually, there is a direct saving to the rates of £2,000 a-year at the expense of the parish surgeons. It might be reasonably supposed that the dispensaries offered a better field than the hospitals for distinguishing between management and maintenance; but here we are met with the same difficulty as before, the cost of the former, without any assignable cause, ranging from 2·3 to 27 and 28 per cent. of the outgoings. It is much to be deplored that in the interests of dispensaries, as well as of hospitals, they are not placed under some kind of organization and supervision by which the money subscribed for their maintenance could be employed to greater advantage. But, as it is with hospitals we are mainly dealing, we will endeavour in future articles to point out some of the causes which have given rise to the increased expenditure, and may possibly suggest some means of rectifying them.

